# Empirical assessment of published effect sizes and power in the recent cognitive neuroscience and psychology literature

**DOI:** 10.1371/journal.pbio.2000797

**Published:** 2017-03-02

**Authors:** Denes Szucs, John P. A. Ioannidis

**Affiliations:** 1 Department of Psychology, University of Cambridge, Cambridge, United Kingdom; 2 Meta-Research Innovation Center at Stanford (METRICS) and Department of Medicine, Department of Health Research and Policy, and Department of Statistics, Stanford University, Stanford, California, United States of America; University of Amsterdam, Netherlands

## Abstract

We have empirically assessed the distribution of published effect sizes and estimated power by analyzing 26,841 statistical records from 3,801 cognitive neuroscience and psychology papers published recently. The reported median effect size was D = 0.93 (interquartile range: 0.64–1.46) for nominally statistically significant results and D = 0.24 (0.11–0.42) for nonsignificant results. Median power to detect small, medium, and large effects was 0.12, 0.44, and 0.73, reflecting no improvement through the past half-century. This is so because sample sizes have remained small. Assuming similar true effect sizes in both disciplines, power was lower in cognitive neuroscience than in psychology. Journal impact factors negatively correlated with power. Assuming a realistic range of prior probabilities for null hypotheses, false report probability is likely to exceed 50% for the whole literature. In light of our findings, the recently reported low replication success in psychology is realistic, and worse performance may be expected for cognitive neuroscience.

## Author summary

Biomedical science, psychology, and many other fields may be suffering from a serious replication crisis. In order to gain insight into some factors behind this crisis, we have analyzed statistical information extracted from thousands of cognitive neuroscience and psychology research papers. We established that the statistical power to discover existing relationships has not improved during the past half century. A consequence of low statistical power is that research studies are likely to report many false positive findings. Using our large dataset, we estimated the probability that a statistically significant finding is false (called false report probability). With some reasonable assumptions about how often researchers come up with correct hypotheses, we conclude that more than 50% of published findings deemed to be statistically significant are likely to be false. We also observed that cognitive neuroscience studies had higher false report probability than psychology studies, due to smaller sample sizes in cognitive neuroscience. In addition, the higher the impact factors of the journals in which the studies were published, the lower was the statistical power. In light of our findings, the recently reported low replication success in psychology is realistic, and worse performance may be expected for cognitive neuroscience.

## Introduction

Low power and selection biases, questionable research practices, and errors favoring the publication of statistically significant results have been proposed as major contributing factors in the reproducibility crisis that is heavily debated in many scientific fields [1–5]. Here, we aimed to get an impression about the latest publication practices in the closely related cognitive neuroscience and (mostly experimental) psychology literature. To this end, we extracted close to 30,000 records of degrees of freedom (df) and *t*-values from papers published between Jan 2011 to Aug 2014 in 18 journals. Journal impact factors ranged from 2.367 (Acta Psychologica) to 17.15 (Nature Neuroscience). The data allowed us to assess the distribution of published effect sizes (D), to estimate the power of studies, and to estimate the lower limit of false report probability (FRP). The text-mining approach we used enabled us to conduct a larger power survey than classical studies.

Low power is usually only associated with failing to detect existing (true) effects, and therefore, with wasting research funding on studies which a priori have a low chance to achieve their objective. However, low power also has two other serious negative consequences: it results in the exaggeration of measured effect sizes and it also boosts FRP, the probability that statistically significant findings are false [5–7].

First, if we use Null Hypothesis Significance Testing (NHST), then published effect sizes are likely to be, on average, substantially exaggerated when most published studies in a given scientific field have low power [6,8] (see **S1A Fig** for the mechanism of effect size exaggeration). This is because even if we assume that there is a fixed true effect size, actual effect sizes measured in studies will have some variability due to sampling error. Underpowered studies will be able to classify as statistically significant only the occasional large deviations from real effect sizes. Conversely, most measured effects will remain under the statistical significance threshold even if they reflect true relationships [9–11]. Effect size inflation is greater when studies are even more underpowered. Consequently, while meta-analyses may provide the illusion of precisely estimating real effects, they may, in fact, estimate exaggerated effects detected by underpowered studies while at the same time not considering unpublished negative findings (see, e.g., [12]).

Secondly, from the Bayesian perspective, the long-run FRP of the NHST framework can be defined as the probability that the null hypothesis (a hypothesis to be “nullified”) is true when we get a statistically significant finding. The long-run True Report Probability (TRP) can be defined as the probability that the alternative hypothesis is true when we get a statistically significant finding [13,5]. Note that the concepts of FRP and TRP do not exist in the NHST framework: NHST only allows for the rejection of the null hypothesis and does not allow for the formal acceptance of the alternative hypothesis. However, here we do not apply NHST but rather, characterize its long-run (“frequentist”) performance from the Bayesian point of view. This approach allows us to talk about true and false null and alternative hypotheses (see more on this in [13,5]).

Computationally, FRP is the number of statistically significant false positive findings divided by the total number of statistically significant findings. TRP is the number of statistically significant true positive findings divided by the total number of statistically significant findings. FRP and TRP can be computed by applying Bayes theorem (see **S1 Text, Section 5** for details).

The overwhelming majority [14] of NHST studies relies on nil–null hypothesis testing [15] where the null hypothesis assumes an exact value. In such cases, the null hypothesis almost always assumes exactly zero difference between groups and/or conditions. For these applications of NHST, FRP can be computed as

$$FRP=\frac{O\alpha }{O\alpha +Power}$$

where O stands for prestudy H_0_:H_1_ odds and α denotes the statistical significance level, which is nearly always α = 0.05. So, for given values of O and α, FRP is higher if power is low. As, in practice, O is very difficult to ascertain, high power provides the most straightforward “protection” against excessive FRP in the nil–null hypothesis testing NHST framework [5–7] (see further discussion of our model in the Materials and Methods section).

Because published effect sizes are likely to be inflated, it is most informative to determine the power of studies to detect predefined effect sizes. Hence, we first computed power from the observed degrees of freedom using supporting information from manually extracted records to detect effect sizes traditionally considered small (d = 0.2), medium (d = 0.5), and large (d = 0.8) [16–18]. Second, we also computed power to detect the effect sizes computed from *t*-values published in studies. Given that many of these published effect sizes are likely to be inflated compared to the true ones (as explained above), this enabled us to estimate the lower limit of FRP [5,13].

## Materials and methods

We extracted statistical information from cognitive neuroscience and psychology papers published as PDF files. We sampled 18 journals frequently cited in cognitive neuroscience and psychology. Our aim was to collect data on the latest publication practices. To this end, we analyzed 4 y of regular issues for all journals published between Jan 2011 to Aug 2014. The time period was chosen to represent recent publication practices (during the closest possible period before the start of data analysis). Particular journals were chosen so as to select frequently cited journals with a range of impact factors from our disciplines of interest.

We categorized ten journals as focused more on (cognitive) neuroscience (Nature Neuroscience, Neuron, Brain, The Journal of Neuroscience, Cerebral Cortex, NeuroImage, Cortex, Biological Psychology, Neuropsychologia, Neuroscience) and five journals focused more on psychology (Psychological Science, Cognitive Science, Cognition, Acta Psychologica, Journal of Experimental Child Psychology). We also searched three more medically oriented journals which are nevertheless often cited in cognitive neuroscience papers so as to increase the representativeness of our sample (Biological Psychiatry, Journal of Psychiatric Research, Neurobiology of Ageing). Journal impact factors ranged from 2.367 (Acta Psychologica) to 17.15 (Nature Neuroscience). Five-year impact factors were considered as reported in 2014 (see **S1 Table**).

When there were fewer than 20 empirical papers in a journal issue, all empirical research reports with any reported *t* statistics were analyzed. When there were more than 20 papers in an issue, a random sample of 20 papers were analyzed merely because this was the upper limit of papers accessible in one query. This procedure sampled most papers in most issues and journals. All algorithms and computations were coded in Matlab 2015b (www.mathworks.com). Initial PDF file text extraction relied on the PdfToolbox Matlab package.

### Data extraction

In summary, a computer algorithm searched through each paper for frequently occurring word and symbol combinations for reporting degrees of freedom and effect sizes provided as Cohen’s *d*. We extracted statistical information about *t* tests and *F* tests (*t*-values, *F*-values, degrees of freedom, *p*-values, and effect sizes). Only *t*-test data is used in this paper, so here we limit data extraction description to *t*-tests.

In psychology and cognitive neuroscience, full *t*-test records are typically reported in the text as, for example, '*t*(df) = x.xx; *p* = y.yy'. D-value reports are often added to these reports as, e.g., '*t*(df) = x.xx; *p* = y.yy; *d* = z.zz'. Hence, in a first text parsing phase, the algorithm opened each PDF file from each journal and identified each point of text which contained a “*t*(” character combination or a “*t*” character. If these characters were identified, then a line of 65 characters were read out from the PDF file starting at the “*t*(” character combination or at the “*t*” character. Spaces between letters and symbols were removed from these lines of text. That is, it did not matter how many spaces separated relevant statistical entries. Lines of text were kept for further analysis if they contained the characters “=“, “<”, or “>” and an additional “p =“, “p<”, or “p>” character combination. This parsing phase identified lines potentially containing independent full *t*-test records. In building this parsing phase, the performance of the algorithm was initially evaluated by reviewing identified lines of text and extracted data from the first 30 papers analyzed for each journal. If specific journals used special characters (as identified by the PdfToolbox package) for typesetting some information (e.g., equation signs), then this was identified and taken into account in the code.

In a second parsing phase, Matlab regular expressions were used to identify full *t*-test records using the templates noted above (e.g., “*t*(df) = x.xx” or “*d* = z.zz”). All text searches were done after converting lines to lowercase characters, so upper- or lowercase differences did not matter in searches.

After data extraction, some error checks were done. First, the algorithm detected a few records twice. This may have happened if for any reason an additional “*t*” appeared within the statistical reporting text (e.g., if researchers used the ‘*t*’ character very close to a statistical record, then that record may have been picked up twice). So, records which had identical statistical information to preceding records were removed. Second, records where negative degrees of freedom (two records) and/or negative *p*-values (one record) were detected were removed. These may have occurred in response to odd character sets or to errors in the text. After cleaning the data, several informal spot-checks were run: hundreds of lines of extracted text were visually compared with the numerical records extracted from the text.

A limitation is that the algorithm only extracted information from the text but not from tables. Further, in order to limit false positive detections (see also later), we restricted our initial search for full *p*-value records, so some reported nonsignificant results and stand-alone *t*-values may have been missed (e.g., *t* < 1; *t* = 0.23). It is important to note that we only assured that our extraction algorithm works fine for the journals and publication years analyzed here. It has not been validated as a more “universal” extraction algorithm like statcheck [19], for example, which we did not know about when starting this project. The extraction algorithm is published as supporting material (**S1 Code**).

### Formal data validation

In a formal validation procedure, we randomly selected 100 papers with *t*-value, *df*, and effect size reports. The selected papers were manually checked for all statistical records. The content of the identified records was then compared to the content of automatically extracted records. This was done to see the accuracy of the computer algorithm and to gather information on the data.

Validation results showed that the automatic extraction algorithm had highly satisfactory performance. The randomly selected papers for validation included 1,478 records of data. The algorithm correctly identified about 95% of *t*-values and degrees of freedom in these records. The algorithm missed only 76 records (5.14%), usually due to atypical punctuation or line breaks within a statistical record. There were no false alarms; that is, all data extracted really belonged to *t*-value records. This is plausible because regular expressions had to fulfill several conditions in order to be identified as potential *t*-test records. For example, it is unlikely that an expression like “*t*(df) = x.x” would stand for anything else than a *t*-value record.

**Fig 1:**
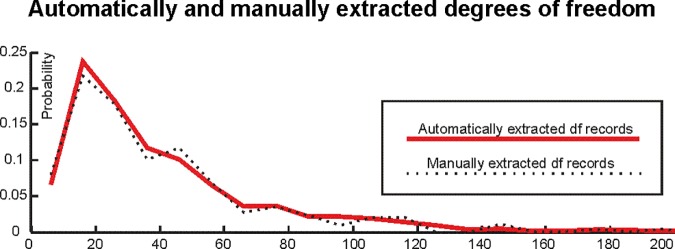
The distribution of automatically and manually extracted degrees of freedom records (“df records”). Note that the distributions are close to overlapping.

The good performance of the extraction algorithm is also reflected in the similarity between the distributions of automatically and manually extracted degrees of freedom shown in **Fig 1** (two-sample Kolgomorov-Smirnov test comparing the distributions: test statistic = 0.04; *p* > 0.127). This suggests that the degrees of freedom distribution underlying our effect size analysis was extracted accurately.

Using the validation data, we found that the overwhelming majority of extracted two sample *t*-test records reported close-to-equal group numbers (median ratio of group numbers = 1). The ratio of the participant numbers in the larger group to the participant numbers in the smaller group was smaller than 1.15 in 77% of records. We also established that with degrees of freedom of ten or less, about 94% of tests were one sample or matched *t*-tests, whereas about 72% of records with higher degrees of freedom were one-sample or matched *t*-tests.

### Computing effect sizes from t tests

*t*-test data was used for effect size, power, and FRP analysis as it is straightforward to estimate effect sizes from published *t*-values. After checks for reporting errors, seven records with degrees of freedom > 10,000 were excluded from analysis as outliers. This left 27,414 potential records. Of these records, 26,841 from 3,801 papers had both degrees of freedom and *t*-values reported. We used this data for the effect size analysis. 17,207 *t*-test records (64.1%) were statistically significant (*p* ≤ 0.05) and 9,634 (35.9%) *t*-test records were statistically nonsignificant (*p* > 0.05). 2,185 *t*-test records also reported Cohen's *d* as a measure of effect size (1,645 records with *p* ≤ 0.05 [75.3%] and 540 records with *p* > 0.05 [24.7%]).

As it is not possible to establish the exact participant numbers in groups for our large sample size, making a few reasonable assumptions is inevitable. First, based on our validation data from 1,478 records, we made the assumption that participant numbers in two-sample *t*-test groups were equal. The number of participants in groups was approximated as the upwards rounded value of half the potential total number of participants in the study, i.e, N_subgroup_ = round_upper_((df+2)/2), where df = degree of freedom. This formula even slightly exaggerates participant numbers in groups, so it can be considered generous when computing power. Second, regarding matched *t*-tests, we assumed that the correlation between repeated measures was 0.5. In such a case, the effect sizes can be approximated in the same way for both one-sample and matched *t*-tests. These assumptions allowed us to approximate effect sizes associated with all *t*-tests records in a straightforward way [20–21]. Computational details are provided in **S1 Text, Section 2.**

Considering the validation outcomes, we assumed that each record with a degree of freedom of ten or less had a 93% chance to be related to a one-sample or matched-sample *t*-test, and other records had a 72% chance to be related to a one-sample or matched-sample *t*-test. Hence, we estimated the effect sizes for each data record with an equation assuming a mixture of *t*-tests where the probability of mixture depended on the degrees of freedom:

$$D=pr(t_{1}|df)∙D_{t1}+pr(t_{2}|df)∙D_{t2}$$

where *pr(t*_1_*|df)* and *pr(t*_2_*|df)* refer to the respective probabilities of one sample and matched *t*-tests (*t*_1_) and independent sample *t* tests (*t*_2_) and D_*t*1_ and D_*t*2_ refer to the respective effect sizes estimated for these tests. df refers to the degrees of freedom.

The power of *t*-tests was computed from the noncentral *t* distribution [22] assuming the above mixture of one-sample, matched-, and independent-sample *t*-tests. Computational details are provided in **S1 Text, Section 3**. Power was computed for each effect size record. (Note that NHST is amalgamation of Fisher’s significance testing method and the Neyman-Pearson theory. However, the concept of power is only interpreted in the Neyman-Pearson framework. For extended discussion, see [23–24]).

First, we calculated power to detect small, medium, and large effect sizes. Power was computed for each extracted statistical record, taking into account the extracted degrees of freedom, a fixed (small, medium, or large) effect size with a significance level of α = 0.05.

Second, we also calculated power to detect the published effect sizes. Importantly, these published effect sizes are likely to be highly exaggerated. Using these exaggerated effect sizes for power calculations will then overestimate power. Hence, if we calculate FRP based on power calculated from published effect size reports, we are likely to estimate the lower limits of FRP. So, we estimated the lower limits for FRP, using the probably highly inflated effect sizes (computed from published *t*-values) to calculate power for various H_0_:H_1_ odds and bias values and with α = 0.05. (The computation of FRP is laid out in detail in **S1 Text, Section 5**.)

In order to get the expected value of FRP for the whole literature, we weighed the FRP computed for each degree of freedom (df) and effect size (D) combination by the probability of that particular (df,D) combination occurring in the research literature and summed the results for all (df,D) combinations:

$${E[FRP]}_{}=\sum_{i=1;j=1}^{i=n;j=m}FRP({df}_{i},D_{j})\;pr({df}_{i},D_{j})$$

An issue worth mentioning is that our model for FRP solely characterizes nil–null hypothesis testing, which is by far the most popular approach to statistics in biomedical science [14]. A very serious drawback of nil–null hypothesis testing is that it completely neglects effect sizes and exclusively directs attention to *p*-values. In addition, it will inevitably detect very small effects as “statistically significant” once statistical power is high enough. However, these small effects can be so close to zero that one could argue that they are practically meaningless. So, from this perspective, if studies with high power detect small effect sizes as statistically significan't this will only increase FRP. Hence, in such cases, paradoxically, increasing power can be thought to lead to increased FRP. This could be taken into account by modifying our basic model described in the introduction as:

$$FRP=\frac{O\alpha +P_{S}∙pr(S)}{O\alpha +P_{S}∙pr(S)+P_{L}∙pr(L)}$$

Where P_S_ stands for power to detect small effects, P_L_ stands for power to detect Large effects, pr(S) and pr(L) stand for the probability of small and large effects, respectively (pr(S) + pr(L) = 1). O stands for prestudy H_0_:H_1_ odds, and α denotes the statistical significance level as before. A difficulty in computing FRP in this way is that the threshold between small and large effects is arbitrary and strongly depends on subjective decisions about what effect size is and is not important. Hence, explicitly modeling the negative impact of detecting very small effect sizes as statistically significant would be fairly arbitrary here, especially as we have collected effect sizes from many different subfields.

Most importantly, factoring in very small but statistically significant effect sizes as false reports into our calculations would only further increase FRP relative to the nill–null hypothesis testing model outlined above. That is, our calculations here really reflect a best-case scenario, the lowest possible levels of FRP when researchers use NHST.

## Results

**Fig 2:**
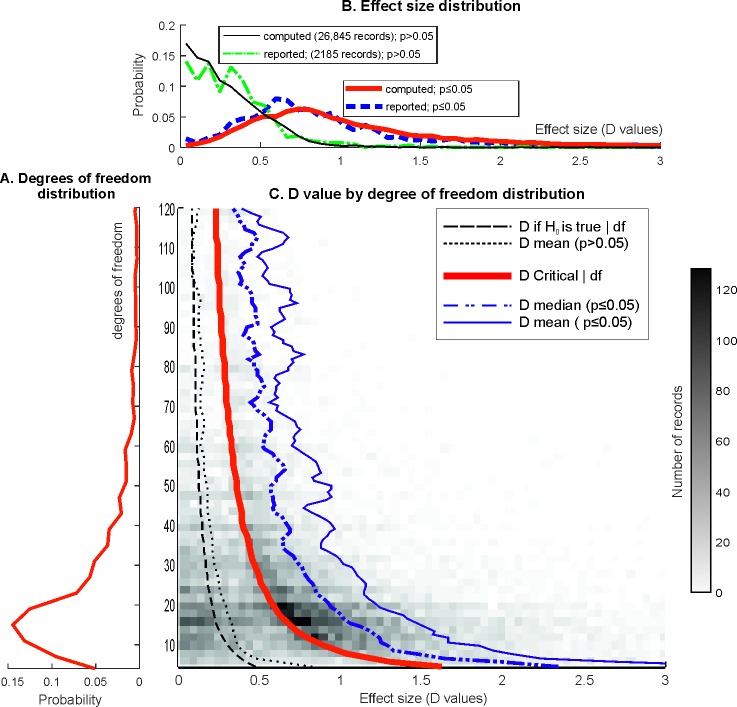
Degrees of freedom and effect size (D-value) distribution in the literature. The histograms are zoomed in for better visibility, but all data were used in calculations. **(A)** Extracted df distribution in all 26,841 records. (The distributions were nearly overlapping for statistically significant and nonsignificant results.) **(B)** The distribution of D-values in statistically significant (*p* ≤ 0.05) and nonsignificant (*p* > 0.05) records in the whole data set (“computed”) and in the subset of data with D-value reports (“reported”). **(C)** The bivariate distribution of D-values and degrees of freedom in the whole data set. The density of statistical records is color coded, as shown by the calibration bar on the right. Curves in the figure are described in a left-to-right order. The leftmost dashed curve shows the expected value of effect sizes if the null hypothesis is true. The dotted curve shows the mean effect size from nonsignificant records in the data (the median was nearly the same). The middle thick continuous red curve denotes the significance threshold with *p* ≤ α where α = 0.05, in terms of D-values. The dotted-dashed blue curve and the rightmost continuous thin blue curve show the median and mean effect sizes only for statistically significant effect sizes, for various degrees of freedom.

The extracted degrees of freedom distributions are shown in **Fig 2A** (degrees of freedom reflect the sample sizes of the studies, e.g., for an independent sample *t*-test, the degrees of freedom are the sample size minus two). The median of degrees of freedom was 20 for statistically significant and 19 for statistically nonsignificant results (mode = 15 for both). During the validation process, we assessed the proportion of one-sample, matched, and two-sample *t*-tests, which enabled us to use a mixture model to compute published effect sizes and power. The distribution of the effect sizes computed from 26,841 *t*-value records showed an excellent match to the effect size distribution determined from 2,185 records where effect size (D)-values were reported **(Fig 2B)**. This suggests that the mixture model we used is likely to well approximate the proportions of one-sample, matched, and two-sample *t*-tests. The computed D-value distribution was more spread out to the right relative to the reported D-value distribution, but both the medians (computed = 0.654; reported = 0.660) and means (computed = 0.938; reported = 0.889) were very similar.

**Fig 2C** shows the bivariate distribution of the 26,841 computed D-values and degrees of freedom and represents the mean and median effect sizes for statistically significant and nonsignificant records and for the whole dataset. Most statistically significant results were reported in the df 10–20 range, and the density of nonsignificant results also increased in this range. The effect size discrepancy between statistically significant and nonsignificant results is clear (medians, 25th and 75th quantiles for statistically significant and nonsignificant D-values, respectively: *d* = 0.932 [0.637–1.458]; *d* = 0.237 [0.106–0.421]).

Assuming 1:1 H_0_:H_1_ odds [5], it is apparent that many statistically nonsignificant results are missing from the data; with 1:1 H_0_:H_1_ odds, a large density of nonsignificant *t*-values could be expected on the left of the significance threshold and an even higher density of nonsignificant than significant results can be expected if H_0_:H_1_ odds are larger than 1 (see the extracted *t*-value distribution in **S2 Fig** and compare the shape of this extracted *t*-value distribution to the expected shapes shown in **S1A and S1B Fig**). Some nonsignificant results are missing because our extraction method could not pick up stand-alone *p*-values. However, the bias towards having mostly significant records in the data (amounting to three quarters of the records here) is also consistent with strong selective reporting biases. Such biases have been demonstrated in distributions of *p*-values reported in abstracts and full texts of biomedical papers [14]. Overall, effect sizes computed from the extracted data are biased towards larger effect sizes. Again, this means that the FRPs we estimate here represent lower limits.

**Fig 3:**
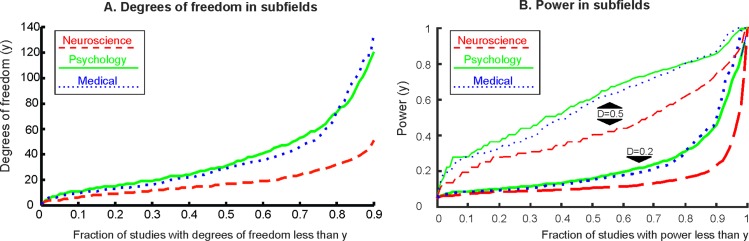
The cumulative distribution of degrees of freedom and power. **(A)** The cumulative distribution of degrees of freedom in subfields. (i.e., the fraction of records with at least a certain level of degrees of freedom by science subfield.) **(B)** The cumulative distribution of power in subfields. The top three lines denote power for an effect size of D = 0.5. The bottom three lines denote power for an effect size of D = 0.2. Power is not shown for effect size of D = 0.8.

For a certain effect size, power is determined by sample size, which determines degrees of freedom. Subfields showed large differences in degrees of freedom with most records having much lower degrees of freedom in cognitive neuroscience than in psychology and medicine (**Fig 3A;** 25th and 75th centiles for all records for cognitive neuroscience journals: df = 10–28; psychology: df = 17–60; medical journals: df = 15–54). Reported effect sizes also differed markedly by subfields (25th and 75th centiles for all records for cognitive neuroscience journals: d = 0.34–1.22; psychology: d = 0.29–0.96; medical journals: d = 0.23–0.91). The larger reported effect sizes in cognitive neuroscience may well be the consequence of effect size exaggeration due to having smaller sample sizes (as shown above) and consequential low power as the following power analyses suggest.

Taking into account the reported degrees of freedom, we computed power (at α = 0.05) for effect sizes, which are traditionally considered small (d = 0.2), medium (d = 0.5), and large (d = 0.8) [16–18]. The cumulative probability of records reaching a certain level of power is shown in **Fig 3B** (for power calculation details see **S1 Text, Section 3**).

Median and mean power for subfields are shown in **Table 1.** Under the assumption that standardized effect sizes are similar in all subfields tested, it is apparent that cognitive neuroscience studies had the lowest level of power. For example, to detect a small true effect (d = 0.2), 90% of cognitive neuroscience records had power < 0.234. This is a much worse chance to detect a true effect than relying on flipping a coin [17].

**Table 1:** Median and mean power to detect small, medium, and large effects in the current study and in three often-cited historical power surveys. The bottom row shows mean power computed from 25 power surveys.

		Small effect		Medium effect		Large effect	
Subfields or other surveys	Records/Articles	Median	Mean	Median	Mean	Median	Mean
**Cognitive neuroscience**	7,888/1,192	0.11	0.14	0.40	0.44	0.70	0.67
**Psychology**	16,887/2,261	0.16	0.23	0.60	0.60	0.81	0.78
**Medical**	2,066/348	0.15	0.23	0.59	0.57	0.80	0.77
**All subfields**	26,841/3,801	0.11	0.17	0.44	0.49	0.73	0.71
**Cohen (1962)**	2,088/70	0.17	0.18	0.46	0.48	0.89	0.83
**Sedlmeier & Gigerenzer (1989)**	54 articles	0.14	0.21	0.44	0.50	0.90	0.84
**Rossi (1990)**	6,155/221	0.12	0.17	0.53	0.57	0.89	0.83
**Rossi (1990); means of surveys**	25 surveys		0.26		0.64		0.85

A comparison to prominent older surveys of power estimates 25 and >50-y-ago showed that median power to detect medium-sized effects has increased slightly in psychology journals but remained about the same for small and large effects (see **Table 1** [16–18]). Power for cognitive neuroscience and for all subfields together was lower than median and mean power reported in 1962, more than half a century ago.

**Fig 4:**
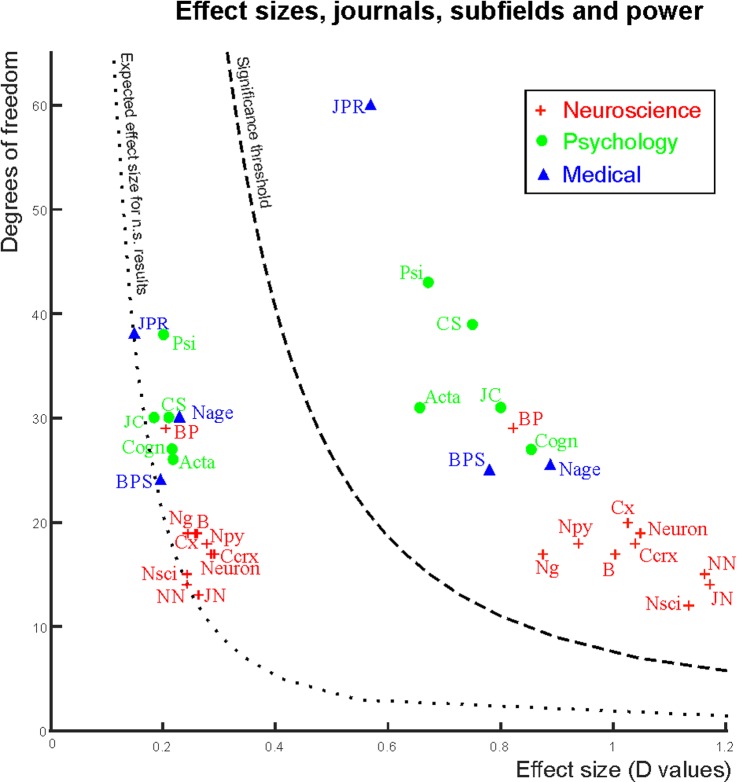
Power in journals and subfields. Median effect sizes and degrees of freedom in the journals analyzed. Red crosses denote cognitive neuroscience journals. Green circles denote psychology journals. Blue triangles denote medically oriented journals. Journal markers to the left of the significance threshold show medians for statistically nonsignificant records (range of medians: d = 0.15–0.29). Journal markers to the right of the significance threshold show medians for statistically significant records (range of medians: d = 0.57–1.17). Journal abbreviations: Neuroscience: Nature Neuroscience (NN), Neuron, Brain (B), The Journal of Neuroscience (JN), Cerebral Cortex (Ccrx), NeuroImage (Ng), Cortex (Cx), Biological Psychology (BP), Neuropsychologia (NPy), Neuroscience (NSci). Psychology: Psychological Science (Psi), Cognitive Science (CS), Cognition (Cogn), Acta Psychologica (Acta), Journal of Experimental Child Psychology (JC). Medically oriented journals: Biological Psychiatry (BPS), Journal of Psychiatric Research (JPR), Neurobiology of Ageing (Nage).

Median degrees of freedom and median effect sizes for each journal are depicted in **Fig 4.** It is apparent that cognitive neuroscience journals report the largest effect sizes but at the same time have the smallest degrees of freedom. Consequently, they also have the lowest power levels assuming similar true effect sizes across fields and are most subject to effect size exaggeration. As a further consequence, journal impact factors negatively correlated with median power because, on the average, cognitive neuroscience journals had the largest impact factors in our sample (correlation for small, medium, and large effect sizes, respectively with 95% accelerated and bias corrected bootstrap confidence intervals [10^5^ permutations]: r = −0.42 [−0.63; −0.09]; −0.46 [−0.71; −0.09]; −0.45 [−0.77; −0.02]).

The somewhat higher power in the journals we classified as more medically oriented was driven by the Journal of Psychiatry Research (JPR in **Fig 4;** median power to detect small, medium and large effects: 0.23, 0.74, 0.86), which includes more behavioral studies than the other two journals we classified as “medical.” These other two journals, more focused on neuroimaging, still performed better than cognitive neuroscience journals and at about the same level as psychology journals (median power to detect small, medium, and large effects: 0.14, 0.53, 0.78).

FRP depends on power (which depends on sample size and effect size), the prestudy odds of true H_0_ to H_1_ data, and on reporting bias [5]. In this context, we use the term “bias” in a general abstract sense as a model parameter as defined by Ioannidis [5]. That is, bias stands for any kind of implicit or explicit technique, manipulation, or error which can result in the outcome that a certain proportion of results which would otherwise be reported as statistically nonsignificant will be reported as statistically significant (see details and mathematical definition in **S1 Text, Section 5**). For example, if the bias parameter equals 0.1, that means that 10% of results which would be reported as statistically nonsignificant in the absence of bias will be now reported as statistically significant. Such bias can easily appear due to data dredging techniques even if formal NHST parameters are maintained [5]. For example, if the main (prespecified) analysis does not yield a formally significant result, investigators may remove or add cases [25], change the model specification [26] and/or data preprocessing parameters in neuroimaging [27], change the statistical analytical method, report on a different outcome, or report a statistically nonsignificant result as significant (e.g., reporting *p* = 0.058 as *p* < 0.05; [28]. Altogether, there are many ways that nonsignificant results may become significant. Frank publication bias (suppression/nonpublication of nonsignificant results), and the rarer fraud with fabrication of nonexistent data or distorting data, yielding significant results will all lead to an excess of reported statistically significant results [29].

**Fig 5:**
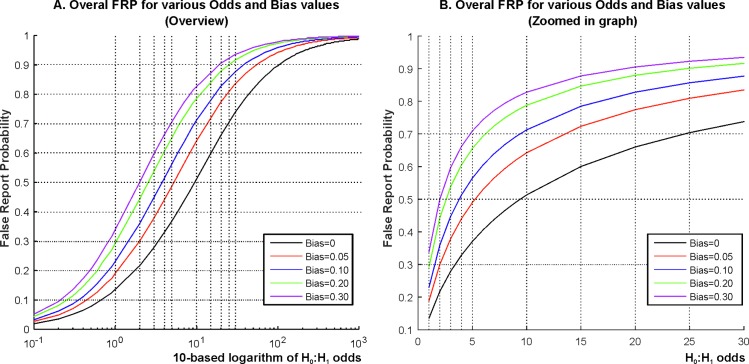
Lower estimates of FRP for various H_0_:H_1_ odds and bias values. **(A)** FRP for a wide range (0.1–1,000) of H_0_:H_1_ odds on a 10-based logarithmic scale (horizontal axis). **(B)** FRP for the 1–30 range. The dotted vertical lines denote the same H_0_:H_1_ odds in both panels for easier comparison (H_0_:H_1_ odds of 1, 2, 3, 4, 5, 10, 15, 20, 25, and 30). Note that the vertical axis begins at 0.1 in Panel B for better visibility. FRP is provided for a range of bias values. For example, bias = 0.1 means that 10% of results that would be reported as statistically nonsignificant in the absence of bias will be now reported as statistically significant. See further elaboration on bias in the text.

The continuous lines in **Fig 5** estimate lower limits for FRP, using the probably inflated effect sizes computed from published *t*-values, for various prestudy H_0_:H_1_ odds and bias values and for α = 0.05. H_0_:H_1_ odds are difficult to determine empirically. First, the nil–null hypothesis is never exactly true. From this perspective, it could be argued that even a very small deviation from the null hypothesis, i.e., a very small effect size, could be considered not only statistically but also practically “significant.” However, very small effect sizes are practically meaningless (see the Materials and Methods section on further elaboration on this). So, when considering H_0_:H_1_ odds, it makes more sense to think about these in the context of effect sizes which could be considered practically meaningful/useful to know about. From such a perspective, it would be unrealistic to assume that most tested hypotheses are really correct (i.e., that they are associated with reasonable effect sizes; [5]); and a recent reanalysis of the Open Science Collaboration replication project [1] also suggests that H_0_:H_1_ odds are likely to be as high as 13:1 (93% true H_0_ situations), at least in psychology [30].

**Fig 5A** represents the lower limits of FRP computed from our data for a wide range of H_0_:H_1_ odds on a 10-based logarithmic scale. Observe that FRP is under 10% only if H_0_:H_1_ odds are smaller than one. In such a case, researchers would mostly come up with correct alternative hypotheses. This is perhaps possible in conservative, very incremental research. On the contrary, in the range of explorative research where H_0_:H_1_ odds are larger than 100, FRP is above 90%.

**Fig 5B** shows FRP zooming into the 1–30 H_0_:H_1_ odds range for better visibility. This range of H_0_:H_1_ odds would still represent a relatively high proportion of correct alternative hypotheses but would keep the ratio of true null hypotheses slightly or moderately higher than the ratio of true alternative hypotheses. Hence, this range of H_0_:H_1_ odds represents a kind of compromise between those who would assume that most null hypotheses are false and those who would assume that most null hypotheses are correct. In the best case of having H_0_:H_1_ odds = 1:1 = 1 and zero bias, FRP is 13.5%. A 10% bias pushes this to 23%. Staying in the optimistic zone when every second to every sixth of hypotheses work out (1 ≤ H_0_:H_1_ odds ≤ 5) and with relatively modest 10%–30% experimenter bias, FRP is 23%–71% (median = 51%). That is, between one- to three-quarters of statistically significant results will be false positives. If we now move into the domain of slightly more exploratory research where even more experimental ideas are likely to be false (5 < H_0_:H_1_ odds < 20; bias = 10%–30%), then FRP grows to at least 60%–91% (median = 77%). Notably, if we consider the recent estimate of 13:1 H_0_:H_1_ odds [30], then FRP exceeds 50% even in the absence of bias.

It is important to note that here we use a single α = 0.05 threshold for FRP calculations because this is the rule of thumb α level used in the science fields we analyzed. That is, even if a record reports, for example, *p* < 0.001, it does not mean that the a priori α level was α = 0.001. Rather, most probably, the result would have been reported as statistically significant as long as the condition p ≤ (α = 0.05) would have been valid (Note that α = 0.05 is an assignment, *p* ≤ α is a test of inequality, and the parentheses are important for correct interpretation. This notation aims to emphasize the crucial difference between the *p*-value and the α level which are often confused. [15]). That is, using a single α = 0.05 threshold here provides the most accurate estimates about the lowest expected limits of FRP in the cognitive neuroscience and psychology literature.

## Discussion

The trustworthiness of statistically significant findings depends on power, prestudy H_0_:H_1_ odds, and experimenter bias [5,7,13]. H_0_:H_1_ odds are inherent to each research field, and the extent and types of biases can vary from one field to another. The distribution of the types of biases may also change within a field if focused efforts are made to reduce some types of major bias (like selective reporting), for example by preregistration of studies. However, power can in principle be easily increased by increasing sample size. Nevertheless, contrary to its importance for the economic spending of research funding, the accurate estimation of effect sizes, and minimizing FRP, our data suggest that power in cognitive neuroscience and psychology papers is stuck at an unacceptably low level. This is so because sample sizes have not increased during the past half-century [16–18]. Results are similar to other fields, such as behavioral ecology where power to detect small and medium effects was 0.13–0.16 and 0.4–0.47, respectively [31].

Assuming similar true effect sizes across fields, we conclude that cognitive neuroscience journals have lower power levels than more psychologically and medically oriented journals. This confirms previous similar inference asserting that FRP is likely to be high in the neuroimaging literature [6,32]. This phenomenon can appear for a number of reasons.

First, neuroimaging studies and other studies using complex and sophisticated measurement tools in general tend to require more expensive instrumentation than behavioral studies, and both data acquisition and analysis may need more time, investment, and resources per participant. This keeps participant numbers low. A related issue is that science funders may have reluctance to fund properly powered but expensive studies.

Second, data analysis is highly technical, can be very flexible, and many analytical choices have to be made on how exactly to analyze the results; and a large number of exploratory tests can be run on the vast amount of data collected in each brain imaging study. This allows for running a very high number of undocumented and sometimes poorly understood and difficult to replicate idiosyncratic analyses influenced by a large number of arbitrary ad hoc decisions. These, in their entirety, may be able to generate statistically significant false positive results with high frequency [27,33–35], especially when participant numbers are low. Hence, sticking to low participant numbers may facilitate finding statistically significant publishable (false positive) results. It is also important to consider that complicated instrumentation and (black box) analysis software is now more available, but training may not have caught up with this wider availability.

Third, in relation to more medical journals, the stakes at risk are probably lower in cognitive neuroscience (no patients will die, at least not immediately), which may also allow for more biased publications. That is, researchers may be more willing to publish less reliable findings if they think that these are not directly harmful. The power failure of the cognitive neuroscience literature is even more notable as neuroimaging (“brain-based”) data is often perceived as “hard” evidence, lending special authority to claims even when they are clearly spurious [36]. A related concern is the negative correlation between power and journal impact factors. This suggests that high impact factor journals should implement higher standards for pre-study power (optimally coupled with preregistration of studies) to assure the credibility of reported results. Speculatively, it is worth noting that the high FRP allowed by low power also allows for the easier production of somehow extraordinary results, which may have higher chances to be published in high impact factor journals [37].

Standardized effect sizes depend on the largeness of effects and the noise level they are embedded in (effect size is larger if signal to noise ratio is better). In behavioral psychology studies, measurement imprecision and variability (e.g., test–retest replicability and reliability, stableness of participant characteristics, etc.) introduce noise. In cognitive neuroscience studies, physiological noise (e.g., various physiological artefacts generated externally or internally to participants) will further contribute to measurement imprecision, while the physiological signals of interest are usually small. Hence, we could expect that measurable standardized effect sizes are in general smaller in cognitive neuroscience than in psychology because both behavioral and physiological noise may contribute to measurements (however, note, as explained before, that due to reliance on NHST, typically only statistically significant exaggerated effect sizes are reported in papers). Were effect sizes really smaller, power would be even worse in cognitive neuroscience relative to psychology than indicated here. Good quality cognitive neuroscience studies may try to counteract physiological noise by increasing trial numbers in individual measurements. A larger number of trials in individuals will then decrease the standard errors of means in these individuals, which may result in smaller group level standard deviations if there is an “ideal” mean measurement value not depending on individuality (but note that individual differences are usually neglected in group studies). This, in turn, will increase group-level *t*-values and effect sizes. Hence, consequences of individual trial numbers have already been taken into account in the calculations reported here when calculating the lower limits of FRP.

Here, we have not explicitly factored in the impact of specific questionable research practices (see, e.g., [26,38]). Rather, we have factored in their potential joint impact through the general “bias” parameter when calculating FRP. Nevertheless, it would be important to see the individual contribution of various data dredging techniques to increasing FRP. For example, researchers may neglect multiple testing correction [39–41]; post hoc select grouping variables [42,26]; use machine-learning techniques to explore a vast range of post hoc models, thereby effectively *p*-hacking their data by overfitting models (http://dx.doi.org/10.1101/078816); and/or liberally reject data not supporting their favored hypotheses. Some of these techniques can easily generate 50% or more false positive results on their own while outputting some legitimate looking statistics [25–26]. In addition, it is also well documented that a large number of *p*-values are misreported, indicating statistically significant results when results are, in fact, nonsignificant [41, 43–45].

With specific respect to functional magnetic resonance imaging (fMRI), a recent analysis of 1,484 resting state fMRI data sets have shown empirically that the most popular statistical analysis methods for group analysis are inadequate and may generate up to 70% false positive results in null data [46,47]. This result alone questions the published outcomes and interpretations of thousands of fMRI papers. Similar conclusions have been reached by the analysis of the outcome of an open international tractography challenge, which found that diffusion-weighted magnetic resonance imaging reconstructions of white matter pathways are dominated by false positive outcomes (http://dx.doi.org/10.1101/084137). Hence, provided that here we conclude that FRP is very high even when only considering low power and a general bias parameter (i.e., assuming that the statistical procedures used were computationally optimal and correct), FRP is actually likely to be even higher in cognitive neuroscience than our formal analyses suggest.

Some limitations need to be mentioned for our study. First, given the large-scale automation, we cannot verify whether the extracted data reflect primary, secondary, or even trivial analyses in each paper. In the absence of preregistered protocols, however, this is extremely difficult to judge, even when full papers are examined. Evaluation of biomedical papers suggests that many reported *p*-values, even in the abstracts, are not pertinent to primary outcomes [3]. Second, some types of errors, such as nondifferential misclassification (measurement error that is not related to the outcome of interest), may lead to deflated effect sizes. However, in the big picture, with very small power, inflation of the statistically significant effects is likely to be more prominent than errors reducing the magnitude of the effect size. Third, given the large scale automated extraction, we did not record information about characteristics of the published studies, e.g., study design. It is likely that studies of different designs (e.g., experimental versus observational studies) may have different distribution of effect sizes, degrees of freedom, and power, even within the same subdiscipline. Hence, we could not take into account the impact of the quality of experimental design on power. Fourth, here we only estimated power for a mixture model of *t*-tests based on the extracted degrees of freedom. Nevertheless, it is very likely that the extracted degrees of freedom give a good indication of participant numbers in studies. These participant numbers would then be strongly correlated with the statistical power of any other analyses done besides *t*-tests. Fifth, we could not extract all nonsignificant relevant *p*-values that are often reported on their own. This biased the observed effect sizes towards larger values. However, this means that the FRPs we computed really reflect lower estimates. Finally, generalizations need to be cautious, since there can be large variability in the extent of these potential biases within a given subfield. Some teams and subfields may have superb, error-proof research practices, while others may have more frequent problems.

In all, the combination of low power, selective reporting, and other biases and errors that have been well documented suggest that high FRP can be expected in cognitive neuroscience and psychology. For example, if we consider the recent estimate of 13:1 H_0_:H_1_ odds [30], then FRP exceeds 50% even in the absence of bias. The low reproducibility rate seen for psychology experimental studies in the recent Open Science Collaboration [1] is congruent with the picture that emerges from our data. Our data also suggest that cognitive neuroscience may have even higher FRP rates than psychology. This hypothesis is worth evaluating with focused reproducibility checks of published studies. Regardless, efforts to increase sample size and reduce publication and other biases and errors are likely to be beneficial for the credibility of this important literature.

Some promising avenues to resolve the current replication crisis could include the preregistration of study objectives, compulsory prestudy power calculations, enforcing minimally required power levels, raising the statistical significance threshold to *p* < 0.001 if NHST is used, publishing negative findings once study design and power levels justify this, and using Bayesian analysis to provide probabilities for both the null and alternative hypotheses [12,26,30,48].

## Supporting information

S1 Fig t value distributions when all negative and positive results are published (df = 22; D = 0.75; α = 0.05 for both panels). **(A)** Illustration of effect size exaggeration due to lack of power. ±t(α) stand for the critical t values. The figure depicts the probability density of t values under a mixture model (Eq 11) assuming a 70% proportion of one-sample t-tests. The thin blue line denotes the probability density of t values if the null hypothesis is true. The thick red line denotes the probability density of t values if the alternative hypothesis is true with an effect size of D = 0.75. Note that because the mixture model assumes a mixture of both one-sample and two-sample t-tests, the probability density curve for t values (under H_1_) is not symmetric. The dashed black line denotes the probability density of t values if in half the data the null hypothesis is true and in the other half the alternative hypothesis is true (ie. The H_0_:H_1_ odds are 1). The little crosses, bars and triangles mark the expected value of absolute t values. Note that these are dramatically different in statistically significant and non-significant data irrespective of whether the null hypothesis is really true or not. Blue bars: the expected t value in data where the null hypothesis is true and the test outcome is non-significant (left bar: true negative) and when the test outcome is significant (right bar: false positive). Red triangles: the expected t value in data where the alternative hypothesis is true and the test outcome is non-significant (left triangle: false negative) and when the test outcome is significant (right triangle: true positive). Black crosses: the expected t values in non-significant (left cross) and significant (right cross) data. Signal detection decision probabilities are shown by α (false positive), 1-α (correct rejection of H_0_), β (false negative) and Power (true positive) in the figure. **(B)** Expected mixture model t value distribution for various H_0_:H_1_ odds(see legend).
(TIF)

S2 Fig The extracted t-value distribution. **(A)** The one dimensional probability density distribution of extracted t-values. **(B)** The two-dimensional t-value by degrees of freedom distribution. The significance threshold [p≤(α = 0.05)] is marked by the white curve. The density of records is shown by the colorbar on the right.
(TIF)

S1 Table Journal information for the three subfields investigated. 5-year journal impact factors used in the study; the number of records in journals; the number of papers by journals and the average number of records per paper.
(DOCX)

S1 Text Supporting Methods. (DOCX)

S1 Data Data in Matlab format. (MAT)

S1 Code Matlab code. (M)

## Abbreviations

D: effect size
df: degree of freedom
fMRI: functional magnetic resonance imaging
FRP: False Report Probability
JPR: Journal of Psychiatry Research
NHST: Null Hypothesis Significance Testing
TRP: True Report Probability
