# Correction: Empirical assessment of published effect sizes and power in the recent cognitive neuroscience and psychology literature

**DOI:** 10.1371/journal.pbio.3001151

**Published:** 2021-03-05

**Authors:** Denes Szucs, John P. A. Ioannidis

Following a re-analysis and validation of all analyses in this article, a very minor discrepancy in few numbers was uncovered, because in a power calculation for two-sample t-tests, (df+2)/2 was used in a formula instead of df+2.

The third sentence of the abstract should read: “Median power to detect small, medium, and large effects was 0.12, 0.46, and 0.78, reflecting no improvement through the past half-century.”

In the sixth paragraph of the results section, the third sentence should read: “For example, to detect a small true effect (d = 0.2), 90% of cognitive neuroscience records had power < 0.242.

The first sentence of the tenth paragraph of the Results section should read: “The somewhat higher power in the journals we classified as more medically oriented was driven by the Journal of Psychiatry Research (JPR in Fig 4; median power to detect small, medium and large effects: 0.24, 0.79, 0.94), which includes more behavioral studies than the other two journals we classified as ‘medical.’”

In the 14^th^ paragraph of the Results section, the fourth sentence on should read: “In the best case of having H_0_:H_1_ odds = 1:1 = 1 and zero bias, FRP is 13.0%. A 10% bias pushes this to 22%. Staying in the optimistic zone when every second to every sixth of hypotheses work out (1 ≤ H_0_:H_1_ odds ≥ 5) and with relatively modest 10%–30% experimenter bias, FRP is 22%–70% (median = 50%). That is, between one- to three-quarters of statistically significant results will be false positives. If we now move into the domain of slightly more exploratory research where even more experimental ideas are likely to be false (5 < H0:H1 odds < 20; bias = 10%–30%), then FRP grows to at least 59%–90% (median = 75%).”

Similarly, corrected power estimates in Table 1 are 0.11, 0.15, 0.42, 0.46, 0.75, and 0.71 in the first row; 0.16, 0.24, 0.64, 0.63, 0.88, 0.84 in the second row; 0.16, 0.24, 0.62, 0.60, 0.87, 0.82 in the third row; and 0.12, 0.18, 0.46, 0.52, 0.78, 0.76 in the fourth row. All other rows and columns remain unchanged.

Please see corrected Table 1.

**Table 1:** Median and mean power to detect small, medium, and large effects in the current study and in three often-cited historical power surveys. The bottom row shows mean power computed from 25 power surveys.

		Small effect		Medium effect		Large effect	
Subfields or other Surveys	Records/Articles	Median	Mean	Median	Mean	Median	Mean
**Cognitive Neuroscience**	**7888/1192**	0.11	0.15	0.42	0.46	0.75	0.71
**Psychology**	**16887/2261**	0.16	0.24	0.64	0.63	0.88	0.84
**Medical**	**2066/348**	0.16	0.24	0.62	0.60	0.87	0.82
**All subfields**	**26841/3801**	0.12	0.18	0.46	0.52	0.78	0.76
**Cohen (1962)**	**2088/70**	0.17	0.18	0.46	0.48	0.89	0.83
**Sedlmeyer and Gigerenzer (1989)**	**54 articles**	0.14	0.21	0.44	0.50	0.90	0.84
**Rossi (1990)**	**6155/221**	0.12	0.17	0.53	0.57	0.89	0.83
**Rossi (1990); means of surveys**	**25 surveys**		0.26		0.64		0.85

A single sentence needs to be corrected in Section 3 of the supplement S1_Text: “Where nt2 = the number of participants in one experimental group (assuming that group sizes are equal as noted above); i.e. nt2 = round_upper_(df+2).”

Please see corrected S1 Text.

## Supporting information

S1 Text Supporting Methods. (DOCX)
