# Supplementary figures and images for: Empirical assessment of published effect sizes and power in the recent cognitive neuroscience and psychology literature
Source: PLoS Biol. 2017 Mar 2;15(3):e2000797. doi: 10.1371/journal.pbio.2000797 (PMC5333800; doi:10.1371/journal.pbio.2000797)

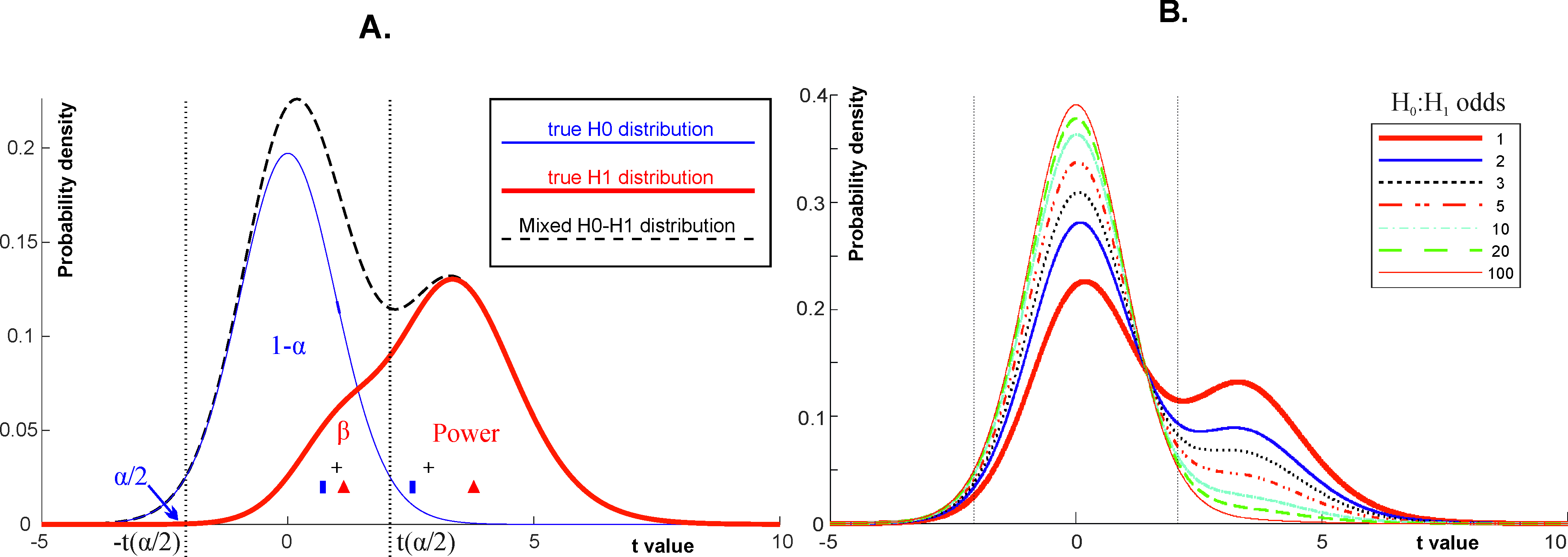

Supplement: S1 Fig — (A) Illustration of effect size exaggeration due to lack of power. ±t(α) stand for the critical t values. The figure depicts the probability density of t values under a mixture model (Eq 11) assuming a 70% proportion of one-sample t-tests. The thin blue line denotes the probability density of t values if the null hypothesis is true. The thick red line denotes the probability density of t values if the alternative hypothesis is true with an effect size of D = 0.75. Note that because the mixture model assumes a mixture of both one-sample and two-sample t-tests, the probability density curve for t values (under H1) is not symmetric. The dashed black line denotes the probability density of t values if in half the data the null hypothesis is true and in the other half the alternative hypothesis is true (ie. The H0:H1 odds are 1). The little crosses, bars and triangles mark the expected value of absolute t values. Note that these are dramatically different in statistically significant and non-significant data irrespective of whether the null hypothesis is really true or not. Blue bars: the expected t value in data where the null hypothesis is true and the test outcome is non-significant (left bar: true negative) and when the test outcome is significant (right bar: false positive). Red triangles: the expected t value in data where the alternative hypothesis is true and the test outcome is non-significant (left triangle: false negative) and when the test outcome is significant (right triangle: true positive). Black crosses: the expected t values in non-significant (left cross) and significant (right cross) data. Signal detection decision probabilities are shown by α (false positive), 1-α (correct rejection of H0), β (false negative) and Power (true positive) in the figure. (B) Expected mixture model t value distribution for various H0:H1 odds(see legend). (TIF) [file pbio.2000797.s001.tif]

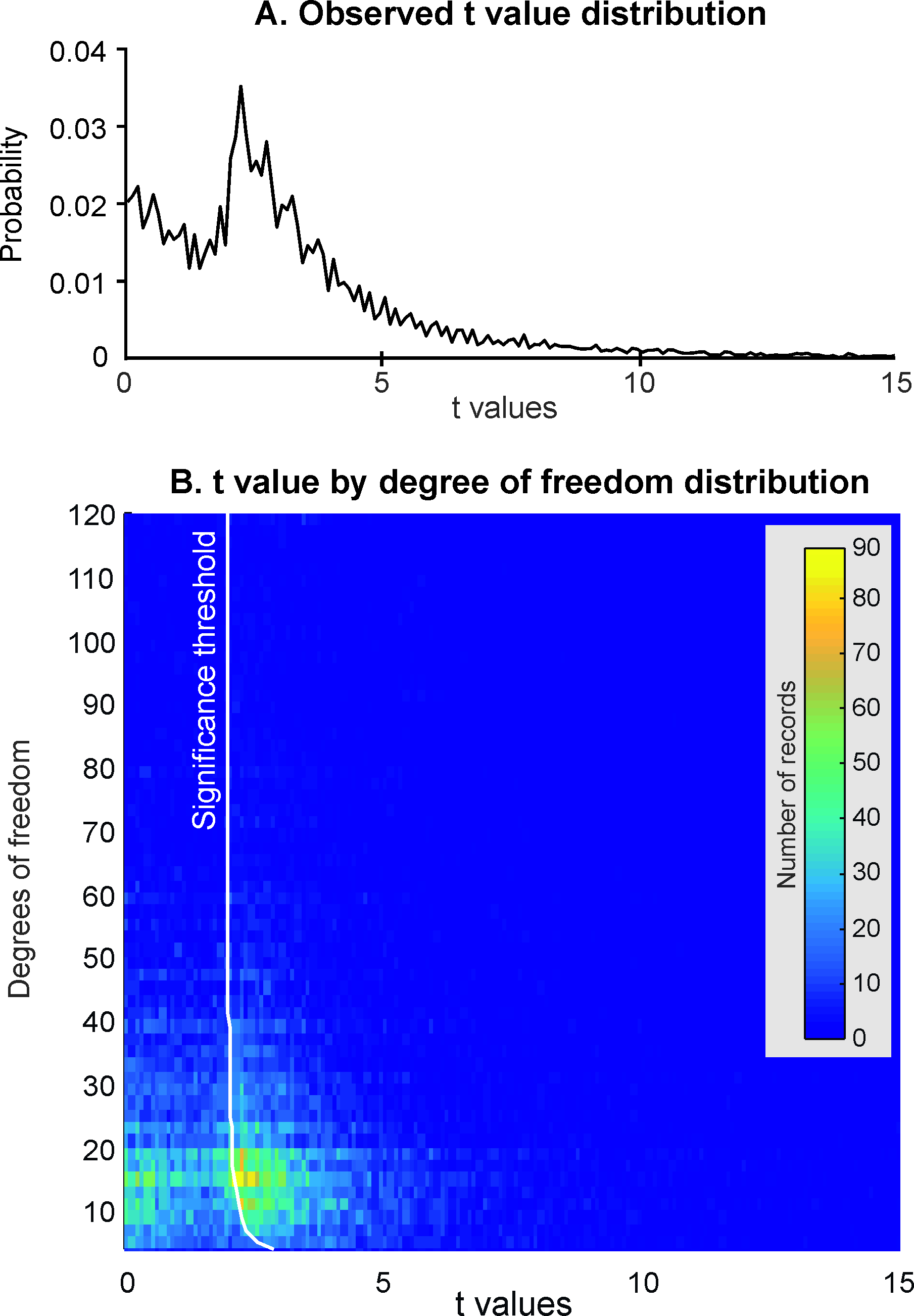

Supplement: S2 Fig — (A) The one dimensional probability density distribution of extracted t-values. (B) The two-dimensional t-value by degrees of freedom distribution. The significance threshold [p≤(α = 0.05)] is marked by the white curve. The density of records is shown by the colorbar on the right. (TIF) [file pbio.2000797.s002.tif]
