# Supplementary material for: Empirical assessment of published effect sizes and power in the recent cognitive neuroscience and psychology literature
Source: PLoS Biol. 2017 Mar 2;15(3):e2000797. doi: 10.1371/journal.pbio.2000797 (PMC5333800; doi:10.1371/journal.pbio.2000797)
Supplement: S1 Table — 5-year journal impact factors used in the study; the number of records in journals; the number of papers by journals and the average number of records per paper. (DOCX) [file pbio.2000797.s003.docx]

**Supplementary Table 1**. shows information about the journals examined.

| **Journals** | **5 year I.F.** | **Records** | **Papers** | **Records/Papers** |
| --- | --- | --- | --- | --- |
| **A. Psychology** |  |  |  |  |
| Psychological Science | 6.16 | 2208 | 387 | 5.7 |
| Cognitive Psychology | 5.508 | 493 | 53 | 9.3 |
| Cognition | 5.088 | 2505 | 316 | 7.9 |
| Acta Psychologica | 3.016 | 769 | 161 | 4.8 |
| JECP | 3.353 | 1913 | 275 | 7.0 |
| **B. Neuroscience** |  |  |  |  |
| Nature Neuroscience | 17.15 | 1212 | 121 | 10.0 |
| Neuron | 16.485 | 834 | 79 | 10.6 |
| Brain | 10.846 | 584 | 75 | 7.8 |
| The Journal of Neuroscience | 7.87 | 5408 | 621 | 8.7 |
| Cerebral Cortex | 8.372 | 1744 | 252 | 6.9 |
| Neuroimage | 6.956 | 971 | 193 | 5.0 |
| Cortex | 5.389 | 1508 | 198 | 7.6 |
| Biological Psychology | 4.173 | 1338 | 205 | 6.5 |
| Neuropsychologia | 4.495 | 2089 | 354 | 5.9 |
| Neuroscience | 3.458 | 1199 | 163 | 7.4 |
| **C. Medical** |  |  |  |  |
| Biological Psychiatry | 10.347 | 1101 | 187 | 5.9 |
| Journal of Psychiatric Research | 4.46 | 468 | 94 | 5.0 |
| Neurobiology of Aging | 5.127 | 497 | 67 | 7.4 |
| **Sums** |  | 26,841 | 3,801 |  |

**Supplementary Table 1. Journal information for the three subfields investigated.** 5-year journal impact factors used in the study; the number of records in journals; the number of papers by journals and the average number of records per paper.
