# Supplementary material for: Empirical assessment of published effect sizes and power in the recent cognitive neuroscience and psychology literature
Source: PLoS Biol. 2017 Mar 2;15(3):e2000797. doi: 10.1371/journal.pbio.2000797 (PMC5333800; doi:10.1371/journal.pbio.2000797)
Supplement: S1 Text — (DOCX) [file pbio.2000797.s004.docx]

**Supplementary Material**

Section 1: Journal information

**Supplementary Table 1**. shows information about the journals examined.

Section 2: Computing effect sizes from t-tests

Effect size calculation for one-sample t-test:

$$D_{t1:one-sample}=\frac{t}{\sqrt{N}}=\frac{t}{\sqrt{df+1}};(1)$$

where *N* stands for the potential total number of participants which is df+1 for one-sample and matched t-tests.

Because, by definition:

$$D= \frac{x-x_{0}}{s};(2)$$

where ‘s’ stands for the sample standard deviation.

So:

$$t_{one-sample}=\frac{x-x_{0}}{s}\sqrt{N}=D\sqrt{N};(3)$$

Effect size calculation for two sample t-test:

$$D_{t2:two-sample}=\frac{2t}{\sqrt{N}}=\frac{2t}{\sqrt{df+2}};(4)$$

where *N* stands for the potential total number of participants which is df+2 for independent sample t-tests.

Because

$$t_{two-sample}= \frac{x_{2}-x_{1}}{s_{12}\sqrt{\frac{1}{n_{1}}+\frac{1}{n_{2}}}};(5)$$

Where n_1_ and n_2_ are the participant numbers in groups and:

$$s_{12}= \sqrt{\frac{\left( n_{1}-1 \right)s_{1}^{2}+ {(n}_{2} -1)s_{2}^{2}}{n_{1}+ n_{2}-2}};(6)$$

And by definition:

$$D=\frac{x_{2}-x_{1}}{s_{12}};(7)$$

Assuming that n_1_ = n_2_ = n_c_ and letting N = n_1_ + n_2_ (ie. N = total number of participants) we get:

$$t_{two-sample}=\frac{D}{\sqrt{\frac{1}{n_{1}}+\frac{1}{n_{2}}}}=\frac{D}{\sqrt{\frac{2}{n_{c}}}}=\frac{D}{\sqrt{\frac{2}{\frac{N}{2}}}}=\frac{D}{\sqrt{\frac{4}{N}}}=\frac{D}{\frac{2}{\sqrt{N}}}=D \frac{\sqrt{N}}{2};(8)$$

So:

$$D_{t2:two-sample}=\frac{2t}{\sqrt{N}}=\frac{2t}{\sqrt{df+2}};(9)$$

For matched-sample t-tests:

$$D_{t1:matched-sample}=\frac{t\sqrt{2\cdot(1-r)}}{\sqrt{N}}=\frac{t}{\sqrt{df+1}};provided r=0.5 ;(10)$$

Section 3: Power calculations

The power of t-tests can be computed from the non-central t distribution assuming an mixture of t tests (see main text for the mixture model we used).

For simpler writing we can first define the cdf for the mixture distribution where the probability of a t value depends on the proportion of one and two-sample t-tests in the data:

MixtureCdf(t,df,pr(t1),D) = pr(t1)*nctcdf(t,df,ϴ_t1_) + [1-pr(t1)]*nctcdf(t,df,ϴ_t2_)$;(11)$

Where

nctcdf stands for the cumulative non-central t probability density function,

t stands for a t value,

df stands for the given degrees of freedom,

pr(t1) stands for the probability of a one-sample or matched t-test.

ϴ_t1_ and ϴ_t2_ are non-centrality parameters dependent on Cohen’s D (Harrison and Brady, 2004):

ϴ_t1_ = D * sqrt(nt1) $;\left( 12 \right)$

Where nt1 = total number of participants; i.e. nt1 = df+1.

ϴ_t2_ = D * ½ * sqrt(nt2) $;\left( 13 \right)$

Where nt2 = the number of participants in one experimental group (assuming that group sizes are equal as noted above); i.e. nt2 = round_upper_((df+2)/2).

Given the above notation, power for a given effect size (D), df and critical t value (t(α)) can be computed as (Harrison and Brady, 2004):

Power(t(α), df, pr(t1), D) = MixtureCdf(t(α),df,pr(t1), D) + (1 - MixtureCdf(t(α),df,pr(t1), D)) $;\left( 14 \right)$

Section 4. Properties of the t-value model and extracted t-values

Based on our mixture model of one-sample and two-sample t-tests we can expect a t value distribution as depicted in **Supplementary Figure 1.** The figure illustrates the mechanism of effect size exaggeration and the importance of H_0_:H_1_ odds.

In **Supplementary Figure 1A.** observe that statistically significant effects always show much larger effect sizes than non-significant effects irrespective of whether they are true positives or false positives. Consequently, a large effect size does not guarantee that results are to be trusted even if they are statistically significant. Effect size exaggeration happens because typically only statistically significant results are reported and these (by definition) must pass the statistical significance threshold. In **Supplementary Figure 1B.** observe that if H_0_:H_1_ odds are high (that is, there are many more true negative than true positive hypotheses to test) then a very high proportion of t values will come from true H_0_ situations. That is, the statistically significant part of the t-value distribution will include a very large number of 'false positive t values' (the 'false positive tail' of the true H_0_ distribution). If H_0_:H_1_ odds are high enough these false positive t-values will outnumber the statistically significant t values coming from the true H_1_ distribution.

The extracted t-value distribution is represented in **Supplementary Figure 2**.

Section 5: False and True Report probability

If we use nil-null Hypothesis Significance Testing (NHST) then the long run False Report Probability (FRP) is the long run probability that the null hypothesis (H_0_) is true when we get a statistically significant finding. The long run True Report Probability (TRP) is the long run probability that the alternative hypothesis (H_1_) is true when we get a statistically significant finding. Computationally, FRP is the number of statistically significant false positive findings divided by the total number of statistically significant findings. TRP is the number of statistically significant truly positive findings divided by the total number of statistically significant findings.

FRP and TRP can be computed by Bayes' theorem. If we take 'sig' to stand for 'statistically significant test outcome' then the total probability of finding a statistically significant result is:

$$pr(\mathrm{sig})= pr\left( \mathrm{sig} | H_{0} \right)*pr\left( H_{0} \right)+ pr\left( \mathrm{sig} | H_{1} \right)*pr\left( H_{1} \right) ;(Eq. 15)$$

Hence, FRP and TRP can be written as:

$$FRP= \frac{\mathrm{pr}\left( \mathrm{sig} | H_{0} \right)*pr\left( H_{0} \right)}{pr(\mathrm{sig})} ;(Eq. 16)$$

$$TRP= \frac{\mathrm{pr}\left( \mathrm{sig} | H_{1} \right)*pr\left( H_{1} \right)}{pr(\mathrm{sig})} ;(Eq. 17)$$

Considering a long run of NHST studies, the long run probability of having a significant test outcome when H_0_ is true is α and the long run probability of having a significant test outcome when H_1_ is true is Power = 1 - β. That is, $\alpha= \mathrm{pr}\left( \mathrm{sig} | H_{0} \right)$ and Power $= p\left( \mathrm{sig} | H_{1} \right)$. Hence, **Eq.16.** and **Eq.17.** can be re-written as:

$$FRP= \frac{\alpha*pr\left( H_{0} \right)}{pr(\mathrm{sig})} ;(Eq. 18)$$

$$TRP= \frac{Power*pr\left( H_{1} \right)}{pr(\mathrm{sig})} ;(Eq. 19)$$

**Eq.16.** and **Eq.17.** can also be expressed in terms of pre-study odds ratios of true H_0_ and true H_1_ situations. For example, we can denote the odds of true H_0_ situations as ‘O’ and write:

$$O= \frac{pr(H_{0})}{pr(H_{1})} ;\left( Eq. 20 \right)$$

We can express pr(H_0_) using the above as:

$$\mathrm{pr}\left( H_{0} \right)= O*pr\left( H_{1} \right) ;\left( Eq. 21 \right)$$

then **Eq.16.** Can be rewritten as:

$$FRP= \frac{\mathrm{pr}\left( \mathrm{sig} | H_{0} \right)*O* pr\left( H_{1} \right)}{\mathrm{pr}\left( \mathrm{sig} | H_{0} \right)*O* p\left( H_{1} \right)+ pr\left( \mathrm{sig} | H_{1} \right)*pr\left( H_{1} \right)} ;(Eq. 17)$$

This can be simplified by $\mathrm{pr}\left( H_{1} \right)$:

$$FRP= \frac{\mathrm{pr}\left( \mathrm{sig} | H_{0} \right)*{Odds}_{H0}}{\mathrm{pr}\left( \mathrm{sig} | H_{0} \right)*{Odds}_{H0}+ pr\left( \mathrm{sig} | H_{1} \right)} ;(Eq. 18)$$

Using α and Power = 1 - β we can write:

$$FRP= \frac{O\alpha}{O\alpha+ Power} ;(Eq. 19)$$

$$TRP= \frac{\mathrm{Power}}{O\alpha+ Power} ;(Eq. 20)$$

$\mathrm{FRP}$ + $\mathrm{TRP}$ = 1; e.g.:

$$\frac{O\alpha}{O\alpha+ Power}+ \frac{Power}{O\alpha+Power}= \frac{O\alpha+Power}{O\alpha+Power}=1 (Eq. 21)$$

Consequently:

$$FRP=1- \mathrm{TRP} \left( Eq. 22 \right)$$

and

$$\mathrm{TRP}=1- \mathrm{FRP} (Eq. 23)$$

Equivalently to the above, we can also express the odds of true H_1_ situations as the ratio of pr(H_1_) and pr(H_0_) and denote it with ‘R’ as in Ioannidis (2005):

$$R= \frac{pr(H_{1})}{pr(H_{0})} ;\left( Eq. 24 \right)$$

$$\mathrm{pr}\left( H_{1} \right)= R*pr\left( H_{0} \right) ;\left( Eq. 25 \right)$$

Substituting **Eq.25.** into **Eq.16.**:

$$TRP= \frac{\mathrm{pr}\left( \mathrm{sig} | H_{1} \right)*R*pr\left( H_{0} \right)}{\mathrm{pr}\left( \mathrm{sig} | H_{0} \right)*p\left( H_{0} \right)+ pr\left( \mathrm{sig} | H_{1} \right)*R*pr\left( H_{0} \right)} ;(Eq. 26)$$

Simplifying:

$$TRP= \frac{\mathrm{pr}\left( \mathrm{sig} | H_{1} \right)*R}{\mathrm{pr}\left( \mathrm{sig} | H_{0} \right)+ R* {Odds}_{H1}} ;(Eq. 27)$$

Using α and Power = 1 - β we can write:

$$TRP= \frac{R*Power}{\alpha+ R*Power} ;(Eq. 28)$$

$$FRP= \frac{\alpha}{\alpha+ R* Power} ;(Eq. 29)$$

**Eq.28.** is equivalent to the one used by Ioannidis (2005) with a slightly different notation.

He defined PPV = $\mathrm{TRP}$; Power = 1-β and equivalently to Eq. 28 he wrote:

$$PPV= \frac{(1-\beta)*R}{\alpha+(1- \beta)*R}= \frac{(1-\beta)*R}{\alpha+R- \beta R} ;(Eq. 29)$$

Ioannidis (2005) also defined Bias, signified by ‘*u*’. On the one hand, bias results in categorizing fraction u (u*(1 - α)) of otherwise true negative results (in case there is no bias) as positive results. On the other hand, bias results in categorizing fraction *u* (uβ = u*(1 - Power)) of otherwise (in case there is no bias) missed true positive results as positive results. That is, bias alters **Eq.28.** as:

$$TRP= \frac{Power*R+u* (1- Power)*R}{\alpha+u* \left( 1- \alpha\right) + Power* R +u* (1- Power)*R} ;(Eq. 30)$$

Using the notation of Ioannidis (2005) this can be rewritten as:

$$PPV= \frac{\left( 1-\beta\right)*R+u\beta R}{\alpha+u\left( 1- \alpha\right)+ \left( 1- \beta\right)* R+u\beta R}= \frac{\left( 1-\beta\right)*R+u\beta R}{\alpha+u-\alpha u+R- \beta R+u\beta R} ;(Eq. 31)$$

Also, notice the relation between $O$ and $R$:

$$O= \frac{p(H_{0})}{p(H_{1})}= \frac{1}{R} ; \left( Eq. 32 \right)$$

Hence,

$$FRP= \frac{\alpha*\frac{1}{R}}{\alpha*\frac{1}{R}+ Power} = \frac{\frac{\alpha}{R}}{\frac{\alpha+Power*R}{R}}= \frac{\alpha}{\alpha+Power*R};(Eq. 33)$$

Similarly:

$$R= \frac{p(H_{1})}{p(H_{0})}= \frac{1}{O} ; \left( Eq. 34 \right)$$

Hence,

$$TRP= \frac{Power* \frac{1}{O}}{\alpha+ Power* \frac{1}{O}} = \frac{\frac{Power}{O}}{\frac{\alpha*O+Power}{O}}= \frac{Power}{\alpha*O+Power} ;\left( Eq. 35 \right)$$

**References**

Harrison D.,A., Brady A.,R., Sample size and power calculations using the noncentral t-distribution. *The Stata journal*. 4, 142-153 (2004).

Fritz, C.,O., Morris, P.,E., Richler, J.,J, Effect size estimates: Current use, calculations and interpretation. *Journal of Experimental Psychology: General.* 141, 2-18 (2012).

Ioannidis, J.P.A. Why most published research findings are false. *PLoS Medicine*. *2,* e124. (2005).

Hunter, J.E & Schmidt F.,L. Methods of meta-analysis: Correcting error and bias in research findings. Newbury park. Sage. (1990).
